# Determining the Potential Roles of Branched-Chain Amino Acids in the Regulation of Muscle Growth in Common Carp (*Cyprinus carpio*) Based on Transcriptome and MicroRNA Sequencing

**DOI:** 10.1155/2023/7965735

**Published:** 2023-06-03

**Authors:** Xianglin Cao, Han Cui, Xinyu Ji, Baohua Li, Ronghua Lu, Yuru Zhang, Jianjun Chen

**Affiliations:** ^1^College of Fisheries, Henan Normal University, Xinxiang 453007, China; ^2^College of Life Science, Henan Normal University, Xinxiang 453007, China

## Abstract

Branched-chain amino acids (BCAAs) can be critically involved in skeletal muscle growth and body energy homeostasis. Skeletal muscle growth is a complex process; some muscle-specific microRNAs (miRNAs) are involved in the regulation of muscle thickening and muscle mass. Additionally, the regulatory network between miRNA and messenger RNA (mRNA) in the modulation of the role of BCAAs on skeletal muscle growth in fish has not been studied. In this study, common carp was starved for 14 days, followed by a 14-day gavage therapy with BCAAs, to investigate some of the miRNAs and genes that contribute to the regulation of normal growth and maintenance of skeletal muscle in response to short-term BCAA starvation stress. Subsequently, the transcriptome and small RNAome sequencing of carp skeletal muscle were performed. A total of 43,414 known and 1,112 novel genes were identified, in addition to 142 known and 654 novel miRNAs targeting 22,008 and 33,824 targets, respectively. Based on their expression profiles, 2,146 differentially expressed genes (DEGs) and 84 differentially expressed miRNA (DEMs) were evaluated. Kyoto Encyclopedia of Genes and Genome pathways, including the proteasome, phagosome, autophagy in animals, proteasome activator complex, and ubiquitin-dependent protein catabolic process, were enriched for these DEGs and DEMs. Our findings revealed the role of *atg5*, *map1lc3c*, *ctsl*, *cdc53*, *psma6*, *psme2*, *myl9*, and *mylk* in skeletal muscle growth, protein synthesis, and catabolic metabolism. Furthermore, miR-135c, miR-192, miR-194, and miR-203a may play key roles in maintaining the normal activities of the organism by regulating genes related to muscle growth, protein synthesis, and catabolism. This study on transcriptome and miRNA reveals the potential molecular mechanisms underlying the regulation of muscle protein deposition and provides new insights into genetic engineering techniques to improve common carp muscle development.

## 1. Introduction

Starvation becomes an environmental stressor for aquatic animals as a result of natural activities, such as environmental and seasonal changes, sexual maturation, disease outbreaks, and food inequalities [1]. Starvation stress frequently leads to a loss of balance in most organs of the fish body, causing poor resistance to disease and pathological discomfort. This in turn negatively affects the quality and yield of the fish, resulting in economic losses [2, 3].

Fish skeletal muscle is the main protein reservoir in the body, generally with a protein content of 15–25%, and is used as a highly plastic tissue that is metabolically active and adapts to metabolic changes [4]. The growth of skeletal muscle is, in essence, a process of proliferation and hypertrophy as the number of muscle fibers and the size of existing muscle fibers increase, which is closely related to the proliferation, differentiation, and fusion of muscle-derived stem cells as well as the deposition of protein in muscle fibers [5]. Branched-chain amino acids (BCAAs; leucine, isoleucine, and valine) are essential for fish and play a critical role in maintaining homeostasis in the internal environment [6]. Fish rely on the mobilization of muscle BCAA catabolism for adaptive regulation of starvation during nutrient deprivation [7, 8], as demonstrated by the impact of muscle BCAA metabolism during fasting in gibel carp or Nile tilapia [9, 10]. BCAAs are the only essential amino acids metabolized at high rates in skeletal muscle; therefore, they are used as anabolizing signals, promoting insulin release, as a substrate for protein synthesis, or in hormonal regulation to influence feeding and blood glucose regulation. They can also directly target the mammalian target of rapamycin 1 (mTOR1) signaling pathway to stimulate skeletal muscle protein synthesis by participating in the phosphorylated eukaryotic translation initiation factor or by inhibiting the ubiquitin-proteasome pathway (UPP) and/or autophagy or lysosomal pathways to inhibit protein hydrolysis, promoting protein turnover [11–14]. The catabolism of BCAAs has been studied extensively. BCAA aminotransferase (BCAT) and BCAA aminotransferase 2 (BCAT2) play key roles in the first step of BCAA catabolism as BCAA-related metabolic enzymes[15], and BCAA dehydrogenase (BCKDH), the rate-limiting enzyme in BCAA catabolism, is modified and inhibited by BCAA dehydrogenase kinase (BCKDK) phosphorylation, which is a key factor in maintaining BCAA homeostasis [16, 17]. Under specific conditions, such as starvation and exercise, the ability of BCAAs to produce succinyl-coenzyme A or acetyl-coenzyme A provides energy to sustain normal life activities [18, 19].

MicroRNAs (miRNAs) play an integral role in the complex process of myogenesis. These small single-stranded RNAs, 21–23 nucleotides long, act as important regulators of various biological processes, interfering with protein translation in the cytoplasm by binding to the 3' UTR region of target mRNAs or causing gene silencing through degradation/cleavage of mRNA transcripts to suppress the expression of target genes [20, 21]. Previous studies have reported that some miRNAs, miR-125a-3p, miR-124-3p, or miR-277, can be involved in the regulation of the skeletal muscle BCAA metabolism by targeting genes [10, 22, 23]. In addition, it has been shown that miR-1, miR-133, and miR-203, which are highly expressed in skeletal muscle, can directly or indirectly target myogenic differentiation genes (*myod*), myogenin (*myog*), and other myogenic factors in muscle-specific myogenic regulatory transcription factors [24, 25]. These genes are involved in muscle development, myoblast proliferation, and differentiation. There are also microRNAs that are significantly altered during myogenesis; for instance, miR-1290 and miR-214 are able to target genes and transcription factors related to muscle protein deposition rate and regulate muscle quality [26, 27].

The development of transcriptomics and other transcriptome sequencing technologies, such as small RNA sequencing, has laid the foundation for genomic research and the identification of genes related to growth and development. Some scholars have conducted relevant discussions on the muscle growth, maintenance of muscle structure and function, and muscle tissue homeostasis of *Mylopharyngodon piceus*, *Danio rerio*, *Salmo salar*, and *Catla catla* through high-throughput RNA sequencing (RNA-Seq) [28–30]. As a lower vertebrate living in water, fish are highly dependent on amino acid catabolism for energy supply [31]. The miRNAs involved in the regulatory mechanisms of BCAAs and their targeted gene relationships in fish muscles under starvation conditions are not well understood; therefore, a more robust approach is needed to discover all potential candidate genes associated with them.

In order to improve the understanding of the regulatory roles of BCAAs, we collected muscle samples from the BCAAs and control groups for mRNA and small RNA sequencing. The genes and miRNA that differed significantly in expression between the two groups were investigated through comprehensive bioinformatics analysis, and the findings were further validated. This study would provide a foundation for further research on the changes of related genes in biological processes, such as muscle protein metabolism, BCAA metabolism, and muscle cell proliferation and differentiation after the addition of BCAAs, as well as the regulatory effect of miRNA as an upstream regulatory factor in the above processes.

## 2. Material and Methods

### 2.1. Sample Collection

In this study, 180 disease-free common carp fishes (20.0 ± 1.50 g; 10.60 ± 0.70 cm) were selected from Xingda Aquaculture Farm, Jinshui District, Zhengzhou City, Henan Province, China. The carp were domesticated in a 300 L volume tank for 2 weeks prior to the formal experiment. Following domestication, 180 fish were randomly divided into two groups with three replicates each: an experimental group and a control group. After 14 days of short-term starvation, 0.1 mL of sterile water and 1.25 g/kg BCAAs (leucine: isoleucine: valine = 2 : 1 : 1) were administered daily using a 1 mL gavage needle. L-isoleucine, L-valine, and L-leucine were purchased from Beijing Solarbio Science & Technology Co., Ltd. (Beijing, China) under the stock keeping unit I0010–25 g, V0010–10 g, and L0011–25 g, respectively, at a purity of ≥98.0%. Sampling was performed two weeks later. The fish (180) were then added to 100 mg/L Tricaine methane sulfonate (MS-222, Shanghai BusChemical Technology Co., LTD.) after anesthesia, and skeletal muscle samples were extracted from the left dorsal tissue between the carp head and dorsal fin, and the samples were then placed in foam boxes containing dry ice and sent to the company of Shanghai Majorbio Bio-pharm Technology Co., Ltd. (Shanghai, China) for sequencing.

In this study, common carp were cultivated to four weeks and maintained at a water temperature of 21.5 ± 1.0°C, the pH was 7.2 ± 0.3, the dissolved oxygen content was >4.5 mg/L, and the ammonia nitrogen level was <0.01 mg/L. Water exchange was one-third of the total volume per day. The light/dark cycle was 12 : 12 h.

### 2.2. Library Construction and High-Throughput Sequencing of the Transcriptome and Small RNA

To resolve the transcript sequences more precisely, RNA was sequenced through Illumina (Megji) sequencing. Total RNA was extracted from carp skeletal muscle tissue samples, and Nanodrop2000 was used to measure the concentration and purity of the proposed RNA. RNA integrity and integrity number were determined by agarose gel electrophoresis and Agilent 2100, respectively. OligodT enrichment was subsequently performed to isolate the mRNA. One-strand cDNA was synthesized by reversing the mRNA template, followed by double-strand synthesis to form a stable double-stranded structure linking the adaptors. After library enrichment and purification of the products (6% Novex TBE PAGE gel; 1.0 mm, 10 wells) and sequencing of the short sequence fragments using the Illumina Novaseq 6000 platform, eukaryotic libraries were constructed using the Illumina TruSeq Small RNA kit. Small RNA statistics require the sequencing of small (18–32 nt) enriched RNA fragments; therefore, the Illumina TruSeq Small RNA kit was used to build the eukaryote library.

### 2.3. Sequence Assembly, Functional Annotation, and Gene Enrichment Analysis

Statistical analysis and quality control of the raw sequence data were performed using FastP, Majorbio Cloud, and FASTX-Toolkit. Subsequently, the clean reads of each sample were compared to the designated reference genome (reference genome source: https://www.ncbi.nlm.nih.gov/genome/?term=Cyprinus_carpio). TopHat2 and HISAT2 software were used for sequence alignment and annotation of transcriptome data. The mapped reads were assembled and compared using Cufflinks and StringTie software to obtain transcripts without annotation information. Functional database annotation analysis was performed on potential new transcripts using NR, Swiss-Prot, Pfam, COG, GO, and KEGG. Bowtie was used to compare the clean reads with the reference genome sequence using the miRBase and Rfam databases to obtain known miRNA and ncRNA annotation information, respectively. The miRDeep2 software was used to predict new miRNAs from unannotated reads. The target gene prediction software, TargetScan and RNAhybrid, were used to predict the miRNA target genes of all known and newly predicted miRNAs, and the target genes were functionally annotated. The GOATOOLS software was used to perform GO enrichment analysis on the genes in the gene set using Fisher's exact test. When the adjusted *p* value was <0.05, the enrichment was considered significant. A KEGG pathway enrichment analysis was performed on the genes or transcripts in the gene set using the R script, and the calculation principle was the same as in the GO functional enrichment analysis.

### 2.4. Examination of Gene Expression Level and Differences

Expression counts were performed for known and new miRNAs and transcripts in each sample; expression was homogenized using transcripts per kilobase of exon model per million mapped reads. Genes and miRNAs that were differentially expressed between the control group (saline administered) and the BCAA group (BCAA administered) were determined using DESeq2, DEGseq, and edgeR software. Differentially expressed miRNAs (DEMs) and differentially expressed genes (DEGs) with fold change (FC) > 2 (upregulation) or FC < 0.5 (downregulation) and a *p* value ≤0.05 were considered to be differentiating significantly between the two groups.

### 2.5. Analysis of miRNA-mRNA Network Interaction

After comparing the target genes predicted by DEMs with DEGs, we constructed the miRNA-target gene network through the Majorbio bioinformatics cloud platform (https://cloud.majorbio.com/page/tools/) according to the miRNA targeted regulation relationship (positive regulation or negative regulation) and miRNA-mRNA interaction relationships.

### 2.6. Validation of DEMs and DEGs by Real-Time Quantitative Polymerase Chain Reaction (qRT-PCR)

Total RNA was extracted using the TRIzol reagent (Invitrogen, USA) after adding 50–100 mg of common carp tissue samples to liquid nitrogen and crushing them with a grinding rod. A NanoDrop spectrophotometer was used to analyze the quality and purity of the total RNA. Samples with ratios of absorbance at 260 nm and 280 nm > 1.8 and RNA concentrations > 100 ng/*μ*L were selected for subsequent experiments. cDNA was synthesized from 2 *μ*L RNA using the Prime Script Reverse Transcriptase Kit (Takara, Japan). The PCR was performed under the following conditions: 37°C for 15 min and 85°C for 5 s, and then cooled to 4°C. The products were stored at -20°C in a refrigerator. qRT-PCR was performed using 25 *μ*L of 2× Ultra SYBR Mixture, 1 *μ*L of each primer, 2 *μ*L of cDNA, and 21 *μ*L of DNase/RNase-free water. The qRT-PCR reaction conditions were as follows: 95°C predenaturation for 10 min, 95°C for 15 s, 65°C for 60 s, and 45 cycles; solubility curve conditions: 95°C for 15 s, 65°C for 60 s, and 97°C for 1 s; and cooling down condition: 37°C for 30 s. Reverse transcription of miRNA first-strand cDNA was performed using the miRcute Plus miRNA First-Strand cDNA Kit (TianGen, China). Subsequent qRT-PCR was performed using the miRcute Plus miRNA qPCR kit (SYBR Green). The miRNA fluorescence quantitative detection reaction system was composed of 5 *μ*L of 2× miRcute Plus miRNA PreMix (SYBR & ROX), 0.2 *μ*L of each primer, 1 *μ*L of miRNA first-strand cDNA, and 3.6 *μ*L of DNase/RNase-free water. In the assay measuring mRNA expression levels, *rpl8* was used as a housekeeping gene to standardize gene expression. miRNA-gene expression levels were assayed using *usb1* as the housekeeping gene to normalize gene expression. All experiments were performed with three biological replicates, and the relative expression levels were calculated using the 2^-*ΔΔ*CT^ method. All primers were designed using Primer Premier 5.0 software and synthesized by Sangon Biotech (Shanghai, China). The synthesized primers were first centrifuged briefly, then dissolved in double-distilled water, diluted tenfold, and placed in a –20°C refrigerator for backup. The relevant primer sequences are listed in Supplementary Table S1.

### 2.7. Statistical Analysis

One-way analysis of variance (ANOVA) and Tukey's test were used to evaluate statistical significance. Differences were considered statistically significant at *p* < 0.05. The data were expressed as mean ± standard deviation (SD). Graphs were created using GraphPad Prism version 5 (GraphPad software Inc., USA).

## 3. Results

### 3.1. Differentially Expressed mRNAs in the Starvation and BCAA Groups

After the transcriptional profiling of two groups with three replicates each, 43.06 gb of clean data were obtained through statistical analysis of the sequencing outcome, with each sample having more than 6.91 gb of clean data and a percentage of Q30 bases above 93.4% (Supplementary Table S2). The clean reads from each sample were compared with the designated reference genome for sequence alignment, with alignment rates ranging from 92.35–93.03% (Supplementary Table S3). A total of 44,526 expressed genes were detected, including 43,414 known and 1,112 novel genes; 77,021 expressed transcripts were detected, including 65,716 known and 11,305 novel transcripts.

**Figure 1:**
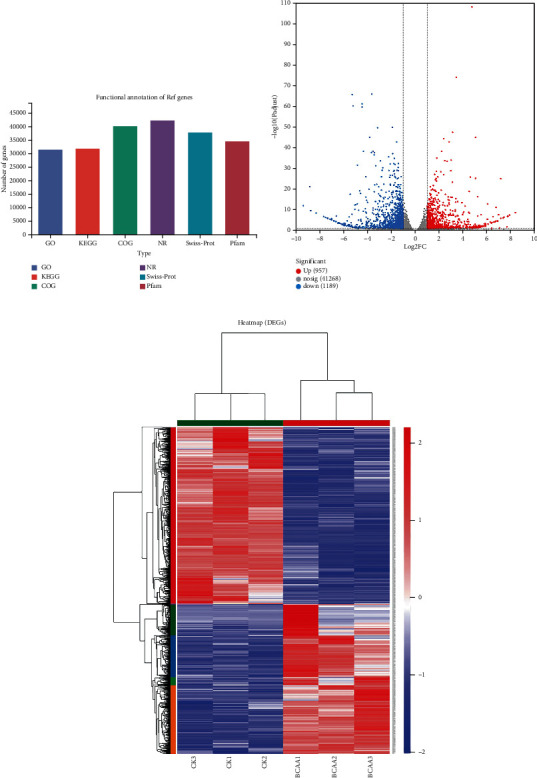
(a) Statistical map of mRNA functional classification. The abscissa indicates the database name, and the ordinate indicates the number or percentage of sequences annotated with that database. (b) Statistical volcano plot showing mRNA expression difference. Red and blue dots in the volcano plot indicate genes with significantly up- or downregulated expression, respectively, and gray dots represent genes with insignificant differences. The *x*-axis shows the log_2_FC, and the *y*-axis shows the log_10_*p* value. (c) Heat map of differential clustering of mRNA expression quantities. Red and blue represent the higher and lower gene expression in this sample, respectively; the left is a dendrogram of gene clustering and a module diagram of subclustering, and the name of the gene is indicated on the right; on the above is a dendrogram with clustering of samples, with name of the sample shown below.

Six functional databases (NR, Swiss-Prot, Pfam, COG, GO, and KEGG) were used to annotate expressed genes and transcripts. The number of annotated genes in the single-gene databases was 42,459 (97.78%) in the NR library, 38,137 (87.84%) in the Swiss-Prot library, 40,342 (92.92%) in the COG library, 31,644 (72.89%) in the GO library, 32,060 (73.85%) in the KEGG library, and 34,726 (79.99%) in the Pfam library. These reads were consistent with unigenes, with a matching rate of 72.89–97.82% (Figure 1(a) and Supplementary Table S4).

Based on the quantitative expression results, differential gene analysis between groups was performed to obtain DEGs between the two groups with a screening threshold of |log_2_FC| ≥ 1.585 and a *p* value <0.05 to investigate the gene expression characteristics of BCAAs supplementation on starving carp skeletal muscle physiological and metabolic changes and other processes. Through this investigation, 2,146 DEGs were identified, of which 957 and 118 were significantly upregulated and downregulated, respectively (Figures 1(b), 1(c)).

**Figure 2:**
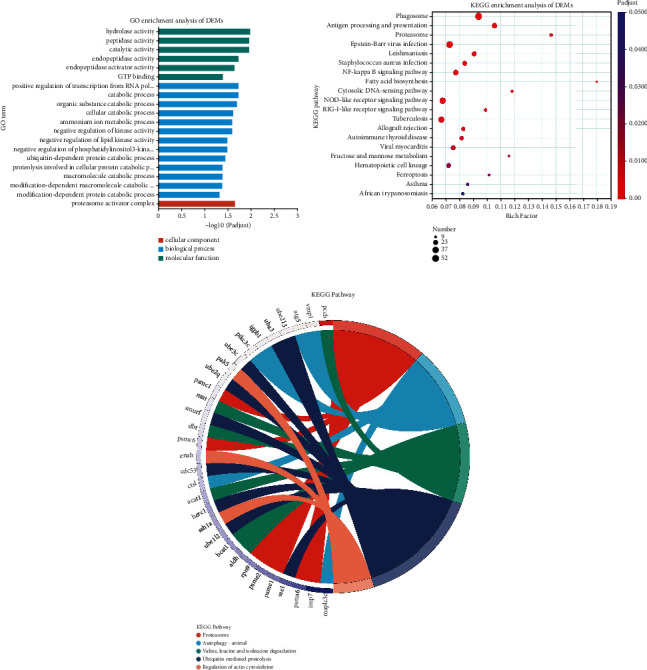
Enrichment and analysis of DEG pathways in GO and KEGG samples. (a) Statistics of the top 20 GO terms enriched for DEGs between two groups. The vertical axis represents GO terms, the horizontal axis represents the rich factors, and the size of the dot represents the number of genes correlated with the GO term. The color of the dot corresponds to different *p-*adjusted ranges, and the smaller the enrichment *p-*adjust, the greater the significance. (b) Top-20 KEGG enrichment results. The vertical axis represents the path name, and the horizontal axis represents the richly factor. The size of the dot indicates the number of genes in this pathway, and the color of the dot corresponds to different *p*-adjusted ranges. (c) KEGG pathway enrichment chord diagram. Red stands for proteasome; blue represents autophagy-animal; green represents valine, leucine, and isoleucine degradation; purple stands for ubiquitin-mediated proteolysis; and pink stands for regulation of actin cytoskeleton in the picture, which denotes a significantly enriched pathway corresponding to differentially expressed genes, with genes on the left. The smaller the log_2_FC, the greater the differential expression of multiple downregulated genes.

To further understand the functions of DEGs, we performed GO and KEGG enrichment analyses. The functions of the DEGs were indicated by GO analysis. A total of 436 terms were enriched, including 133 molecular functions (MF), 32 cellular components (CC), and 269 biological processes (BP). Peptidase activity and GTP binding were identified as major MFs by transcriptome GO enrichment; the major BPs were modification-dependent protein catabolic process, proteolysis involved in cellular protein catabolic process, ubiquitin-dependent protein catabolic process, and positive regulation of transcription from the RNA polymerase II promoter involved in the cellular response to chemical stimuli; the main CC was the proteasome activator complex (Figure 2(a) and Supplementary Table S5).

Through KEGG analysis, 330 pathways were enriched for DEGs in the skeletal muscle of the control and BCAA groups. We selected the 20 representative pathways with the highest enrichment rate of DEGs for subsequent analysis. (Figure 2(b) and Supplementary Table S6). The metabolic pathways enriched in both DEG groups were mainly related to the proteasome, phagosome, fructose and mannose metabolism, and fatty acid biosynthesis. In the KEGG enrichment table, pathways related to other muscle growth pathway were identified, and DEGs associated with these biological functions were screened (Figure 2(c)). These DEGs were *atg5*, *map1lc3c*, *vmp1*, *igbp1*, and *pik3c3*, which were shown to be associated with the autophagy pathway (*p* < 0.05). Proteolytic processes, such as the lysosomal pathway and the ubiquitin-proteasome pathway, were enriched for *psma6*, *psme2*, *lmp7*, *uba3*, *ube1l2*, *sae1*, and *cdc53*. Additionally, biological processes, including valine, leucine, and isoleucine degradation, were significantly affected, and the related genes in the process, *bcat1*, *acat1*, *dbt*, and *aldh*, were significantly (*p* < 0.05) downregulated. In addition, the expression levels of multiple muscle growth-related genes, such as *rheb*, *irs2*, *myh9s*, *myl9*, *mylk*, *mef2c*, *fosb*, and *mapk12a*, were significantly different (*p* < 0.05).

### 3.2. Differentially Expressed miRNAs in the Starvation and BCAA Groups

**Figure 3:**
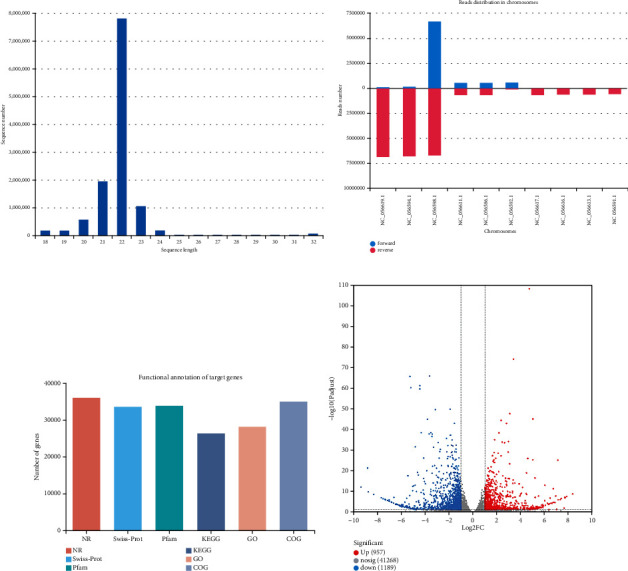
Analysis of DEMs. (a) Statistical chart of a useful read length distribution for small RNA sequencing. The abscissa represents the sequence length, and the ordinate indicates the number. (b) Statistical map of miRNA chromosome distribution. The abscissa is the chromosome number, and the ordinate is the number of sequenced reads that are located on the chromosome. (c) Target gene functional annotation summary statistical chart. The abscissa is the individual database name, and the ordinate is the number of annotations for the target gene in each database. (d) miRNA expression difference volcano plot. The ordinate value is the amount of expression of that gene in the treated sample. The red dots represent significantly upregulated miRNAs, the blue dots represent significantly downregulated miRNAs, the gray dots represent significantly differential miRNAs, and the point with a greater degree of deviation from the diagonal indicates a greater expression difference between the two samples.

A total of 80.65 M raw reads were obtained by deep small RNA sequencing of two sets of samples, and the raw reads of each sample reached above 12.22 M, with the percentage of Q30 based at 87.9% and above (Supplementary Tables S7 and S8). After the raw sequencing data of each sample were quality-controlled to obtain clean reads, the 18–32 nt reads were screened as useful reads for subsequent analysis, according to the sRNA length characteristics (Figure 3(a)). The number of reads that were subsequently compared ranged from 9,831,207 to 13,472,097 (Supplementary Table S9). Additionally, the distribution on each chromosome was determined, and it was observed that most of the small RNAs were distributed on chromosomes NC_056619.1, NC_056594.1, and NC_056598.1 (Figure 3(b)). In miRBase, clean reads were homologous sequences, whereas new miRNA predictions were performed for reads that were not compared for homology. A total of 796 miRNAs were detected in all samples, corresponding to 36,044 targets; 142 known miRNAs targeted 22,008 targets, and 654 novel miRNAs targeted 33,824 targets (Supplementary Tables S10.). These target genes were functionally annotated in six major databases (NR, Swiss-Prot, EggNOG, GO, KEGG, and Pfam), and the number of genes annotated was 36,005 (99.89%) in the NR library, 33,572 (93.14%) in the Swiss-Prot library, 34,964 (97.00%) in the COG library, 28,128 (78.04%) in the GO library, 26,304 (73.85%) in the KEGG library, and 33,795 (93.76%) in the Pfam library. These reads were consistent with data unigenes, with a matching rate of 72.98–99.89% (Figure 3(c) and Supplementary Table S11).

Through differential analysis of samples from BCAAs and control groups, 84 DEMs were obtained, of which 45 were significantly upregulated, and 39 were significantly downregulated (*p* < 0.05) (Figure 3(d) and Supplementary Tables S12).

**Figure 4:**
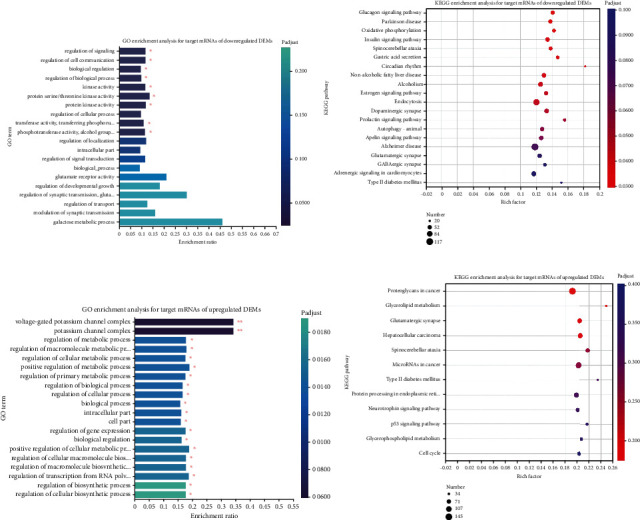
Enrichment analysis of GO and KEGG pathways on the basis of DEMs. (a) GO enrichment analysis for target mRNAs of downregulated DEMs. The vertical axis represents the GO term, and the horizontal axis represents the richly factor. The size of the dots represents the number of genes in this GO term, and the color of the dots corresponds to different *p-*adjusted ranges. (b) KEGG enrichment analysis for target mRNAs of downregulated DEMs. The vertical axis indicates the KEGG pathway name, the horizontal axis indicates the rich factors, the size of the dot indicates how many genes are in this KEGG pathway, and the color of the dot corresponds to different *p-*adjusted ranges. (c) GO enrichment analysis for target mRNAs of upregulated DEMs. The vertical axis represents the GO term, and the horizontal axis represents the richly factor. The size of the dots represents the number of genes in this GO term, and the color of the dots corresponds to different *p-*adjusted ranges. (d) KEGG enrichment analysis for target mRNAs of upregulated DEMs. The vertical axis indicates the KEGG pathway name, the horizontal axis indicates the rich factors, the size of the dot indicates how many genes are in this KEGG pathway, and the color of the dot corresponds to different *p-*adjusted ranges.

Among the 39 downregulated DEMs, miRNAs with significant expression differences included ccr-mir-135c, ccr-mir-194, and ccr-mir-192 (*p* < 0.05). Through the target genes, 600 GO terms were enriched for the above miRNAs (Figure 4(a) and Supplementary Table S13). In other words, these significantly downregulated DEMs are all involved in the regulation of developmental growth, protein kinase activity, protein serine/threonine kinase activity, and regulation of biological processes through target genes. A KEGG enrichment analysis was performed on DEMs to further assess how miRNAs affect mRNAs. The findings revealed that these miRNAs may be associated with the regulation of the apelin signaling pathway, autophagy in animals, the insulin signaling pathway, oxidative phosphorylation, and other pathways (Figure 4(b) and Supplementary Table S14).

Among the 54 upregulated miRNAs, the most significant expression levels were observed for ccr-miR-203b-3p, ccr-miR-725, ccr-miR-184, and ccr-miR-205 (*p* < 0.05). The top 20 GO terms that were enriched for the upregulated DEMs mainly include regulation of cellular biosynthetic processes, regulation of macromolecule biosynthetic processes, and regulation of cellular metabolic processes. The KEGG enrichment analysis showed that these upregulated miRNAs could be associated with the cell cycle, the p53 signaling pathway, and protein processing in the endoplasmic reticulum (Figures 4(c) and 4(d) and Supplementary Tables S15 and S16).

### 3.3. Interactive Network for DEMs and DEGs

**Figure 5:**
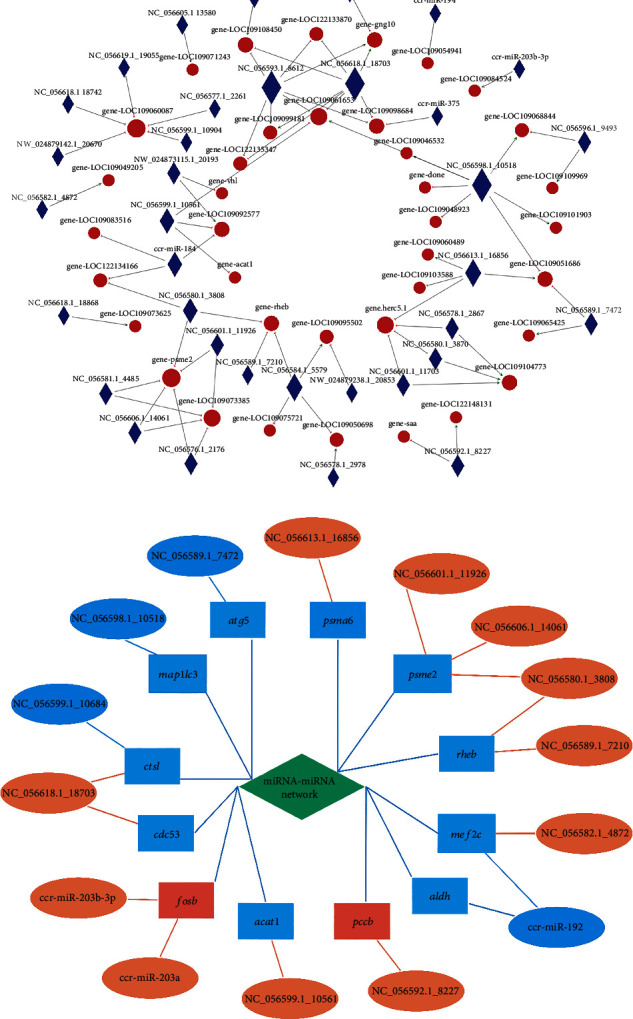
Coexpression network of DEMs and DEGs. (a) Statistical network diagram of miRNA-mRNA relationships. Through the miRNA and mRNA interaction relationship, miRNA and target gene action pairs (miRNA-gene pairs) were visualized in a network form. Circular nodes represent genes, and diamond nodes represent miRNAs in the figure; internode lines (directed) represent the presence of targeting regulation between the two, green arrows represent positive regulation, and red short erector bars represent negative regulation. (b) Association network diagram of skeletal muscle growth-related genes and miRNAs. The ellipses in the figure represent the DEMs, the rectangles represent the DEGs, orange and red represent their expression quantities upregulated, and the opposite is blue that represents the expression quantity downregulated. Blue lines indicate positive correlations, and orange lines indicate negative correlation.

According to the results of differential expression analysis and functional analysis, network analysis for crosstalk between DEGs and DEMs was constructed to involve in regulating the growth of carp skeletal muscle. The results of interactive network showed that 957 DEGs were upregulated, and 1,189 DEGs were downregulated, of which 38 DEGs were targeted by 35 DEMs, forming 75 pairs of miRNA-mRNA relationship. Additionally, 51 pairs showed negatively associated expression patterns, and 24 pairs showed positively associated expression patterns (Figure 5(a)). Further screening results showed that after the addition of BCAAs, DEMs such as NC_056618.1_18703, NC_056582.1_4872, NC_056589.1_7210, and NC_056601.1_11926 might be able to regulate metabolism, BCAA metabolism, and genes related to muscle proliferation and differentiation, including *ctsl*, *cdc53*, *psma6*, *psme2*, *acat1*, *mef2c*, and *rheb*, that were involved in the regulation of skeletal muscle growth in hungry common carp (Figure 5(b)).

### 3.4. Candidate miRNAs and mRNA Expression Validation

**Figure 6:**
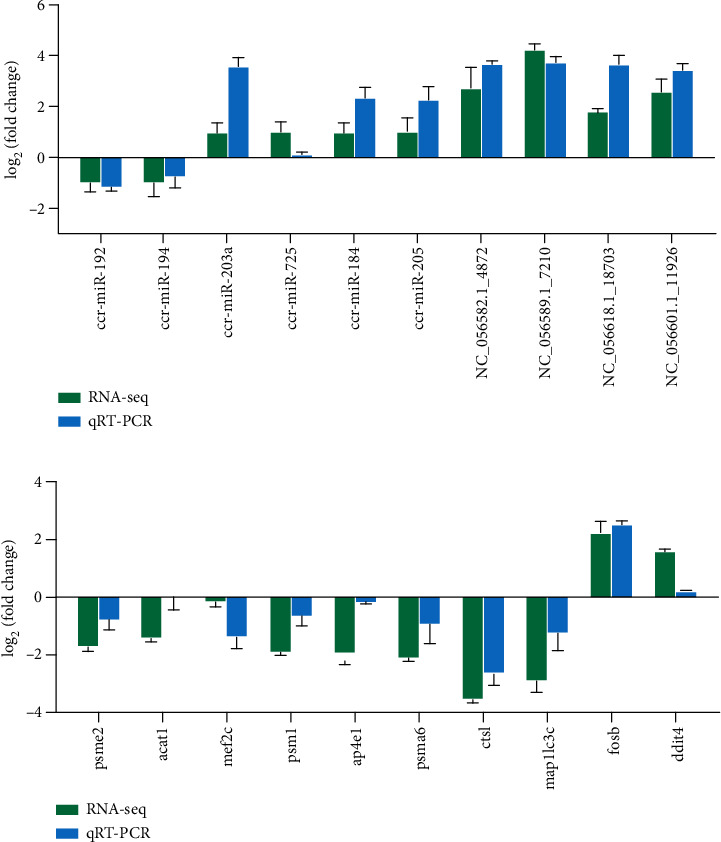
Transcriptome and small RNA sequencing data and real-time quantitative PCR (qRT-PCR) correlation validation map. Validation of the expression of mRNAs (a) and miRNAs (b) through qRT-PCR. The *y*-axis shows the relative expression (log_2_FC). Bars represent the standard deviation of the three biological replicates.

To confirm the DEG findings, ten muscle growth-related genes, *psme2*, *acat1*, *mef2c*, *psm1*, *ap4e1*, *psma6*, *ctsl*, *map1lc3c*, *fosb*, and *ddit4*, were examined by qRT-PCR. Additionally, nine other miRNAs that had positively or negatively correlated expression patterns with genes associated with muscle protein metabolism and growth, including miR-192, miR-194, miR-203a, miR-184, miR-205, NC_056582.1_4872, NC_056589.1_7210, NC_056618.1_18703, and NC_056601.1_11926, were investigated. qRT-PCR validation of the selected DEGs and DEMs indicated the same expression pattern as revealed by RNA-Seq and small RNA-Seq (log_2_FC), despite the different levels of expression (Figure 6). This consistency with the sequencing results confirmed the reliability of the sequencing data.

## 4. Discussion

Skeletal muscle, a key metabolic site in fish, plays a critical role in adaptive regulation in response to dietary nutrient deficiencies [7]. Under fasting conditions, some fish use body proteins as energy substrates after consuming large amounts of body fat, when the amount of functional substrate amino acids rises compared to essential and nonessential amino acids [31]. Among them, BCAAs can participate in several cellular signal cascades affecting anabolism and catabolism and maintain the physiological dynamic balance of fish under the condition of food shortage [11]. In this study, we used transcriptome sequencing combined with small RNA sequencing technology to analyze which fish skeletal muscle growth-related mRNA and miRNA changes the body phenotype after short-term fasting by adding BCAAs. DEG and DEM spectra of the continuous starvation group and the group with BCAAs added after starvation showed that these DEGs could participate in the proteasome, phagosome, and fatty acid biosynthesis through protein catabolic process, proteasome activator complex, and ubiquitin-dependent protein catabolic process. The target genes of DEMs are enriched in autophagy, insulin signaling pathway, oxidative phosphorylation, cell cycle, and p53 signaling pathway, as well as participate in the regulation of developmental growth, macromolecule biological process, and cellular metabolic process. The results of these experiments indicated that fasting results in degradation of protein and amino acids in common carp and a negative balance of protein turnover. However, BCAAs participate in the regulation of the ubiquitin-protease system and the autophagy-lysosomal system, alleviating the decomposition of muscle proteins under starving conditions.

In our study, the expression of some miRNAs changed significantly under different treatments. This may reflect the diversity and importance of miRNAs playing their own functions in the nutritional regulation of the organism. Previous studies have shown that some miRNAs tend to have multiple targets, and they regulate the physiological activities of the body by targeting diverse signaling pathways [21]; therefore, the identification of miRNA target genes is an important step in understanding miRNA function. The results indicated that the target gene *rheb* was coregulated by NC_056589.1_7210 and NC_056580.1_3808, which were negatively correlated. In addition, the expression of *rheb* was clearly downregulated. Existing reports show that as an upstream regulator of mTOR/raptor, *rheb* overexpression can inhibit IRS1-phosphatidylinositol-3-kinase- (PI3K-) Akt signal transduction, which affects skeletal muscle production [32]. Apart from this, as a typical p53-responsive miRNA, miR-194 promotes cellular senescence in mouse embryonic fibroblasts by targeting *dnmt3a* for inhibition [33]. Similarly, miR-192 participates in the primary activation of P53 when cells are subjected to DNA damage, whereas miR-22 further downregulates P21 and activates E2F1 to induce apoptosis [34]. Furthermore, overexpression of miR-192 could target the myogenic modulator retinoblastoma 1 to inhibit myogenic differentiation of sheep satellite cells [35]. Expression of both miR-192 and miR-194, belonging to the miR-192 family of miRNAs, was significantly reduced in the BCAA group. Additionally, NC_056582.1_4872 was significantly upregulated and predicted to target *mef2c*, which showed a negative correlation. *mef2c* is a key upstream activating factor of *myoc* and participates in the regulation of skeletal muscle growth via downstream *myod1 and myog* [36]. Furthermore, several muscle growth-associated genes, including *myh9s*, *myl9*, *mylk*, *fosb*, and *mapk12a*, were significantly upregulated in the BCAA group. Existing studies have proved that activation of mapk12a participates in the heterodimerization of *myod* and E proteins, leading to *myod* activation and myogenic cell differentiation [37]. *myh9* encodes the heavy chain of nonmuscle myosin IIA, a ubiquitously expressed cytoplasmic myosin that regulates the actin cytoskeleton, cell migration, and PI3K/Akt/mTOR signaling in cells [38]. Some studies suggest that *myl9* and *mylk* may play important roles in skeletal muscle growth and strength [39]. The Fos gene family is capable of encoding leucine zipper proteins involved in the formation of the transcription factor complex AP-1 [40]. Several studies have shown that overexpression of *fosb* in the Fos gene family promotes myogenic cell differentiation and proliferation and inhibits apoptosis. *fosb* plays an active role in regulating processes such as myoblast fusion into myotubes [41, 42]. Overall, these findings indicate that the addition of BCAAs may affect skeletal muscle differentiation during short-term starvation stress in common carp to some extent.

UPP contains a network of enzymes that are responsible for maintaining cellular protein homeostasis [13]. In this study, the expression of ubiquitin-activated enzymes, *uba3*, *ube1l2*, *sae1*, and *cdc53*, was significantly downregulated. UBA3 is a catalytic protein with E1-like activity in the NEDD8 pathway that activates NEDD8/RUB1 and covalently links to cullin proteins to promote ubiquitination, playing a key role in protein degradation [43]. *ube1l2* sequence is 40% identical to *ube1* and can also activate ubiquitin in an ATP-dependent manner; both were significantly downregulated in this study [44]. SAE1 is a heterodimeric small ubiquitin-related modifier that plays a critical role in SUMOylation [45]. While CDC53 acts as a scaffolding protein in the E2/E3 core complex, regulating the cell cycle and growth through ubiquitination [46], *cdc53* is a predicted target gene of NC_056618.1_18703, with a negative correlation existing between their expression. As a result, NC_056601.1_11926, NC_056606.1_14061, NC_056580.1_3808, NC_056613.1_16856, NC_056618.1_18703, and NC_056601.1_11926 may contribute to fish proteolysis, which deserve to be explored in more detail.

As expected, BCAAs were also able to alleviate the muscle protein breakdown process by regulating autophagy. Several proteolysis-associated genes were identified in this study, such as the autophagy pathway-related genes, *atg5*, *map1lc3c*, *vmp1*, *pik3c3*, and *igbp1*, and genes related to proteolytic processes, such as the lysosomal pathway, including *psma6*, *psme2*, and *lmp7*, which are implicated in the ubiquitin-proteasome pathway, whose expression was significantly downregulated. Among these, MAP1LC3C, as a member of the LC3 subfamily, stabilizes the lipidation of LC3C/GABARAP-L2 in the early steps of autophagosome formation and helps maintain the unidirectional flow of autophagosomes to lysosomes. Furthermore, atg5, a key regulator of autophagy, regulates the expression of its own genes that participate in autophagy in a feedback inhibitory loop [47]. Vmp1 regulates ptdins3k activity at the phagophore membrane through interaction with becn1, whose activity favors the production of 3-phosphate phosphatidylinositol (PtdIns3P) and affects the association of autophagy-related proteins (including Atg16L1) with the phagophore membrane. Moreover, Vmp1 is also able to regulate autophagy induction through Vmp1-Beclin 1 interaction [48]. PIK3C3 can convert phosphatidylinositol to PtdIns3P, which maintains autophagy or autophagy and endosomal transport in macrophages and is utilized by starvation-induced autophagy [49]. PSMA6 is a proteasomal subunit of the 20S catalytic core complex, and its genetic polymorphisms can affect proteasomal activity [50, 51]. The proteasome activator T-006 activates the PKA/Akt/mTOR or p70S6 kinase pathway and enhances proteasome activity by upregulating LMP7 expression [52]. In the predicted results of the targeting relationship, NC_056601.1_11926, NC_056606.1_14061, NC_056580.1_3808, and NC_056613.1_16856 were found to simultaneously target and inhibit *psme2*, resulting in a significant downregulation of its expression. Previous experiments have demonstrated that PSME1/2 reduces *β*-catenin protein levels, leading to a restriction of Wnt/*β*-catenin signaling. During nutrient deprivation, the key Wnt signaling proteins *β*-catenin and Disheveled are targeted for autophagic degradation via LC3 to affect cell growth, proliferation, and apoptosis [53, 54].

Our findings imply that *bcat1*, *acat1*, *dbt*, and *aldh*, as related enzymes in the BCAA catabolic process, were significantly downregulated. Notably, *acat1* was predicted to be a potential target gene of NC_056599.1_10561. Both ACAT2 and ACAT1 belong to the acetyl coenzyme A acetyltransferase (ACAT) family. ACAT1 participates in acetyl acyl coenzyme A ketogenesis and the ketolytic and isoleucine degradation pathways. In this study, *aldh*, one of the predicted targets of miR-192, was significantly downregulated in the experimental group. In addition to regulating BCAA catabolism, *aldh* plays an important role in skeletal muscle homeostasis and is involved in the regulation of aldehyde metabolism and the regenerative capacity of skeletal muscle. DBT is involved in the composition of the E2 subunit of the BCKDH complex, while BCAT-catalyzed transamination and BCKDH-catalyzed oxidative decarboxylation are components of the BCAA metabolism. The differential expression of *dbt* can influence the rate-limiting function of BCKDH in regulating the catabolic balance of BCAAs. BCAT1 and BCAT2, two isozymes of BCAT, are involved in catalytic transamination during BCAA catabolism. BCAT1, the cytoplasmic aminotransferase of BCAAs, is involved in the maintenance of BCAA concentrations and regulation of mitochondrial biogenesis.

Fish can increase muscle mass by replenishing new muscle fibers and can also increase the size of existing muscle fibers to promote skeletal muscle growth [5]. Our study found that the supplementation of BCAAs can act separately from these two aspects in conjunction with skeletal muscle growth. Although no significant changes in myogenic-related genes and miRNAs were found, there were significant differences in myodifferentiation-related genes and miRNAs between the experimental group and the control group, which was necessary for the growth of skeletal muscle tissue [4]. It has been reported that the addition of BCAAs can promote the synthesis of protein by activating the signal of skeletal muscle mTORC1 or regulating the initiation of transcription or translation. From the perspective of maintaining protein's positive metabolic balance, it is very important to reduce the activation of protein catabolism in skeletal muscle under nutritional stress [12, 13]. In our research, we found that there were significant differences in genes and miRNAs between the two groups, which were related to two proteolytic systems used for protein degradation, namely, ubiquitin-proteasome system and autophagy-lysosome system. At the same time, we speculate that with the passage of time, the catabolic pathway of protein is inhibited, and the body can not effectively recover amino acids. This leads to the corresponding adjustment of amino acid homeostasis in organisms, which makes the genes related to BCAA catabolism significantly downregulated, and makes the three amino acids themselves become the exchange substrates, thus achieving steady-state balance. Overall, dynamic changes in the expression of these genes and the mechanisms regulated by them may be experienced by the organism to maintain normal physiological activities in response to nutrient deprivation, followed by the addition of BCAAs. These findings provide evidence that BCAAs can inhibit autophagy and ubiquitination in carp skeletal muscle under nutritional stress, prevent the degradation of muscle proteins, participate in the regulation of muscle cell differentiation to some extent, and maintain physiological homeostasis under food deprivation.

## 5. Conclusions

The study involved transcriptome and small RNA sequencing of carp skeletal muscle under nutritional stress and then after the addition of BCAAs. Many DEGs and DEMs were identified in this study; several important DEGs were selected to explore the effects of BCAA addition on starvation-induced genes and miRNAs related to skeletal muscle growth. Sequencing analysis revealed significant changes in the expression of protein metabolism genes and muscle differentiation genes in skeletal muscle of fasted common carp and those of carp fed BCAAs, expanding the knowledge of the regulatory effects of BCAAs on muscle growth mechanisms. In addition, the identified large-scale gene and small RNA list lays the foundation for the possible regulation of related miRNAs and their target genes in fish skeletal muscle, greatly enriching molecular resources and helping improve yield and sustainable aquaculture.

## Data Availability

The authors confirm that the data supporting the findings of this study are available within the article and its supplementary materials.

## Abbreviations

BCAA: Branched-chain amino acids
miRNAs: MicroRNAs
mRNA: Messenger RNA
DEGs: Differentially expressed genes
DEMs: Differentially expressed miRNAs.

## Additional Points

*Highlights*. (1) Branched-chain amino acids (BCAAs) may regulate carp skeletal muscle growth. (2) miRNAs are involved in the BCAA-mediated protein metabolism and muscle differentiation in carp skeletal muscle. (3) Under nutrient stress, BCAAs inhibit autophagy and ubiquitination in carp skeletal muscle.

## Supplementary Materials

Supplementary Materials The supplementary table includes S1-16. Supplementary Table S1: RT-qPCR primers for mRNAs and miRNAs. Supplementary Table S2: statistics of transcriptome sequencing data. Supplementary Table S3: statistics of mRNA alignment results. Supplementary Table S4: mRNA functional classification statistics. Supplementary Table S5: GO enrichment analysis (DEMs). Supplementary Table S6: KEGG enrichment analysis (DEMs). Supplementary Table S7, S8: small RNA raw data statistical table; small RNA quality control data statistical table. Supplementary Table S9: statistical table of small RNA comparison results. Supplementary Table S10: miRNA target gene prediction statistics table. Supplementary Table S11: miRNA annotation profile statistics. Supplementary Table S12: miRNA differential gene number statistics. Supplementary Table S13: GO enrichment analysis (target mRNAs of downregulated DEMs). Supplementary Table S14: KEGG enrichment analysis (target mRNAs of downregulated DEMs). Supplementary Table S15: GO enrichment analysis (target mRNAs of upregulated DEMs). Supplementary Table S16: KEGG enrichment analysis (target mRNAs of upregulated DEMs).
