# Supplementary material for: Determining the Potential Roles of Branched-Chain Amino Acids in the Regulation of Muscle Growth in Common Carp (Cyprinus carpio) Based on Transcriptome and MicroRNA Sequencing
Source: Aquac Nutr. 2023 Jun 3;2023:7965735. doi: 10.1155/2023/7965735 (PMC10257547; doi:10.1155/2023/7965735)
Supplement: Supplementary Materials — The supplementary table includes S1-16. Supplementary Table S1: RT-qPCR primers for mRNAs and miRNAs. Supplementary Table S2: statistics of transcriptome sequencing data. Supplementary Table S3: statistics of mRNA alignment results. Supplementary Table S4: mRNA functional classification statistics. Supplementary Table S5: GO enrichment analysis (DEMs). Supplementary Table S6: KEGG enrichment analysis (DEMs). Supplementary Table S7, S8: small RNA raw data statistical table; small RNA quality control data statistical table. Supplementary Table S9: statistical table of small RNA comparison results. Supplementary Table S10: miRNA target gene prediction statistics table. Supplementary Table S11: miRNA annotation profile statistics. Supplementary Table S12: miRNA differential gene number statistics. Supplementary Table S13: GO enrichment analysis (target mRNAs of downregulated DEMs). Supplementary Table S14: KEGG enrichment analysis (target mRNAs of downregulated DEMs). Supplementary Table S15: GO enrichment analysis (target mRNAs of upregulated DEMs). Supplementary Table S16: KEGG enrichment analysis (target mRNAs of upregulated DEMs). [file 7965735.f1.docx]

**Supplementary materials**

Supplementary Table S1: RT-qPCR primers for mRNAs and miRNAs

Sequences of the primers used in this experiment.

| Primer | Acc. Num. | Oligonucleotide sequence (5′→3′) |
| --- | --- | --- |
| *psme2* | XM_019089518.2 | F: GATCTCACAGCTGGACAACTT  R: AGAAACAGTGATGCAGGTCTC |
| *acat1* | XM_042733015.1 | F: CTTTTGCCGTCCCCAAGTTT  R: GACACTGCTCCTCCGTTGAT |
| *mef2c* | XM_042733110.1 | F: ATCGCCCTCATCATCTTTAACAGCA  R: TGTCACAGCCGTTTAGACCCTT |
| *psm1* | XM_019099743.2 | F: AAAGCGGAAAGAAGGGTGG  R: CTTGGTGCGGGTGTTGGTA |
| *ap4e1* | XM_019124298.1 | F: CCACAACATCACTTACGCAATC  R: TCAAGGCTTTCAAACCAA |
| *psma6* | XM_042739995.1 | F: CAAAGCCATCAGTCAGAGC  R: TGTCAGCGTAATGTCCAGTC |
| *ctsl* | XM_042774144.1 | F: CCAGTGCTGCCAGTCTTT  R: CAGGTCTGCTTTCGGTGA |
| *map1lc3c* | XM_042738226.1 | F: CGCCGAAATGATGGACTTCAC  R: TCTACTGTTGCCTCTGGACG |
| *fosb* | XM_042743892.1 | F: AGAAGAGCAACGACACCACAA  R: TTGTACGGCTGGTTCTTGGTT |
| *ddit4* | XM_019093485.2 | F: CTATCAAGACCAGCCTCCC  R: CTTCACAGTCAAACCCACCACGGAG |
| *rpl8* | XM_042731504.1 | F: TGTTGAGCATCCCTTCGG  R: GGTCTITGTTCCACGCAGT |
| ccr-miR-192 | MIMAT0026250 | F: AUGACCUAUGAAUUGACAGCC |
| ccr-miR-194 | MIMAT0026252 | F: UGUAACAGCAACUCCAUGUGGA |
| ccr-miR-203a | MIMAT0026260 | F: GUGAAAUGUUUAGGACCACUUG |
| ccr-miR-184 | MIMAT0023348 | F: GGATGGACGGAGAACTGATAAGGGC |
| ccr-miR-205 | MIMAT0026263 | F: CTCCTTCATTCCACCGGAGTCTGC |
| NC_056582.1_4872 |  | F: ACGTGTCCGATCCTGCAGAGTT |
| NC_056589.1_7210 |  | F: CGTTGTCTCAATCCTGTATGAATTCGGA |
| NC_056618.1_18703 |  | F: TGCCCTGAACTGTTCTTCCCAGAG |
| NC_056601.1_11926 |  | F: GCAGAATTGTGCCTGGACATCTGTAA |
| *usb1* |  | F: ACGGCGCTTCGGCAGCACATATAC |

Reverse primer: AACGCTTCACGAATTTGCGT.

Supplementary Table S2 Statistics of transcriptome sequencing data

| Sample | Raw reads | Raw bases | Clean reads | Clean bases | Error rate(%) | Q20(%) | Q30(%) | GC content(%) |
| --- | --- | --- | --- | --- | --- | --- | --- | --- |
| BCAA3 | 54638358 | 8.25E+09 | 53273530 | 7.71E+09 | 0.0249 | 98.02 | 94.3 | 50.12 |
| BCAA2 | 49004336 | 7.4E+09 | 47630322 | 6.94E+09 | 0.0258 | 97.64 | 93.4 | 50.34 |
| BCAA1 | 50772962 | 7.67E+09 | 49574518 | 7.26E+09 | 0.0247 | 98.1 | 94.5 | 50.29 |
| CK3 | 48159900 | 7.27E+09 | 47191500 | 6.91E+09 | 0.0245 | 98.18 | 94.65 | 50.36 |
| CK2 | 50671290 | 7.65E+09 | 49269394 | 7.19E+09 | 0.0249 | 98.01 | 94.29 | 50.32 |
| CK1 | 49718604 | 7.51E+09 | 48230660 | 7.04E+09 | 0.0257 | 97.7 | 93.56 | 50.15 |

Supplementary Table S3 Statistics of mRNA alignment results

| Sample | Total reads | Total mapped | Multiple mapped | Uniquely mapped |
| --- | --- | --- | --- | --- |
| BCAA3 | 53273530 | 49334158(92.61%) | 2596804(4.87%) | 46737354(87.73%) |
| BCAA2 | 47630322 | 44027927(92.44%) | 2568244(5.39%) | 41459683(87.04%) |
| BCAA1 | 49574518 | 46076895(92.94%) | 2491888(5.03%) | 43585007(87.92%) |
| CK3 | 47191500 | 43902530(93.03%) | 2383734(5.05%) | 41518796(87.98%) |
| CK2 | 49269394 | 45778417(92.91%) | 2461749(5.0%) | 43316668(87.92%) |
| CK1 | 48230660 | 44541974(92.35%) | 2476006(5.13%) | 42065968(87.22%) |

Supplementary Table S4 mRNA functional classification statistics

|  | Expre_Gene number（percent） | Expre_Transcript number（percent） | All_Gene number（percent） | All_Transcript number（percent） |
| --- | --- | --- | --- | --- |
| GO | 31644(0.7289) | 43567(0.663) | 40288(0.6762) | 62045(0.6223) |
| KEGG | 32060(0.7385) | 49839(0.7584) | 40067(0.6724) | 70849(0.7106) |
| COG | 40342(0.9292) | 61935(0.9425) | 50475(0.8471) | 88288(0.8856) |
| NR | 42450(0.9778) | 64627(0.9834) | 55031(0.9236) | 94508(0.9479) |
| Swiss-Prot | 38137(0.8784) | 59099(0.8993) | 46929(0.7876) | 83417(0.8367) |
| Pfam | 34726(0.7999) | 54939(0.836) | 40612(0.6816) | 75657(0.7589) |
| Total_anno | 42469(0.9782) | 64651(0.9838) | 55080(0.9244) | 94563(0.9485) |
| Total | 43414(1.0) | 65716(1.0) | 59584(1) | 99698(1) |

Supplementary Table S5 GO enrichment analysis (DEMs)

| GO enrichment analysis (DE mRNAs) | | | | | | | | |
| --- | --- | --- | --- | --- | --- | --- | --- | --- |
| **Number** | **GO ID** | **Term Type** | **Description** | **Ratio_in_study** | **Ratio_in_pop** | **Pvalue** | | **Padjust** |
| 103 | GO:0008233 | MF | peptidase activity | 103/1563 | 1709/40668 | 0.00000809710885884 | | 0.0110555419549 |
| 87 | GO:0005525 | MF | GTP binding | 87/1563 | 1473/40668 | 0.0000779793455807 | | 0.0411471013514 |
| 46 | GO:0019941 | BP | modification-dependent protein catabolic process | 46/1563 | 656/40668 | | 0.000124579763809 | 0.0478084281478 |
| 50 | GO:0051603 | BP | proteolysis involved in cellular protein catabolic process | 50/1563 | 726/40668 | 0.0000826925594292 | | 0.0413207578325 |
| 46 | GO:0006511 | BP | ubiquitin-dependent protein catabolic process | 46/1563 | 642/40668 | 0.0000672513540039 | | 0.0370291803089 |
| 5 | GO:1901522 | BP | positive regulation of transcription from RNA polymerase II promoter involved in cellular response to chemical stimulus | 5/1563 | 10/40668 | 0.0000178618142767 | | 0.0184745866708 |
| 4 | GO:0008537 | CC | proteasome activator complex | 4/1563 | 6/40668 | 0.0000306388143898 | | 0.0228241144372 |

Supplementary Table S6 KEGG enrichment analysis (DEMs)

| KEGG enrichment analysis(DE mRNAs) | | | | | | | |
| --- | --- | --- | --- | --- | --- | --- | --- |
| **Number** | **Pathway id** | **Description** | **Ratio_in_study** | **Ratio_in_pop** | **Pvalue** | **Padjust** |  |
| 52 | map04145 | Phagosome | 52/1524 | 554/40352 | 2.26097204011e-9 | 7.4612077323 |  |
| 18 | map03050 | Proteasome | 18/1524 | 123/40352 | 9.03389453654e-7 | 0.0000993728 |  |
| 35 | map04064 | NF-kappa B signaling pathway | 35/1524 | 452/40352 | 0.0000580138560392 | 0.00319076208 |  |
| 11 | map00051 | Fructose and mannose metabolism | 11/1524 | 95/40352 | 0.000919680361216 | 0.01785261877 |  |
| 13 | map04216 | Ferroptosis | 13/1524 | 128/40352 | 0.00114531982705 | 0.0209975301 |  |
| 9 | map00071 | Fatty acid degradation | 9/1524 | 98/40352 | 0.0118990048172 | 0.1354024686 |  |

Supplementary Table S7 Small RNA Raw Data Statistical Table

| Sample | Raw reads | Raw bases | Error rate(%) | Q20(%) | Q30(%) | GC content(%) |
| --- | --- | --- | --- | --- | --- | --- |
| BCAA3 | 12837226 | 9.63E+08 | 0.0282 | 96.63 | 91.42 | 48.83 |
| BCAA2 | 12340914 | 9.26E+08 | 0.0348 | 93.16 | 87.9 | 49.13 |
| BCAA1 | 13382651 | 1E+09 | 0.0286 | 96.4 | 91.18 | 46.36 |
| CK3 | 13080008 | 9.81E+08 | 0.0299 | 95.73 | 90.29 | 47.43 |
| CK2 | 16795375 | 1.26E+09 | 0.0278 | 96.77 | 91.82 | 47.35 |
| CK1 | 12217830 | 9.16E+08 | 0.0342 | 93.6 | 87.97 | 47.4 |

Supplementary Table S8 Small RNA Quality Control Data Statistical Table

| Sample | Clean reads | Clean bases | Error rate(%) | Q20(%) | Q30(%) | GC content(%) | Useful reads(18nt-32nt) |
| --- | --- | --- | --- | --- | --- | --- | --- |
| BCAA3 | 12729785 | 2.8E+08 | 0.0249 | 98.25 | 94.08 | 36.92 | 12359919 |
| BCAA2 | 12166589 | 2.68E+08 | 0.0245 | 98.37 | 94.5 | 36.73 | 11772043 |
| BCAA1 | 13194364 | 2.91E+08 | 0.0253 | 98.05 | 93.64 | 37.71 | 12689565 |
| CK3 | 12936661 | 2.86E+08 | 0.0246 | 98.31 | 94.42 | 36.96 | 12441298 |
| CK2 | 16597399 | 3.65E+08 | 0.0244 | 98.43 | 94.54 | 36.7 | 16015365 |
| CK1 | 12109223 | 2.68E+08 | 0.025 | 98.14 | 93.96 | 36.63 | 11639149 |

Supplementary Table S9 Statistical table of small RNA comparison results

| Sample | Total reads | Total mapped | Mapped reads (+) | Mapped reads (-) |
| --- | --- | --- | --- | --- |
| BCAA3 | 12359919 | 10379913 | 9465219 | 9680374 |
| BCAA2 | 11772043 | 9920741 | 9166857 | 9321747 |
| BCAA1 | 12689565 | 10480302 | 9600306 | 9737291 |
| CK3 | 12441298 | 10440913 | 9557265 | 9759511 |
| CK2 | 16015365 | 13472097 | 12385690 | 12598700 |
| CK1 | 11639149 | 9831207 | 9069718 | 9208569 |

Supplementary Table S10 miRNA target gene prediction statistics table

| Type | miRNA | miRNA with Target | | Target |
| --- | --- | --- | --- | --- |
| Known miRNA | 142 | 142 | 22008 | |
| Novel miRNA | 654 | 654 | 33824 | |
| Total | 796 | 796 | 36044 | |

Supplementary Table S11 miRNA annotation profile statistics

| Type | target number | target percent |
| --- | --- | --- |
| NR | 36005 | 0.9989 |
| Swiss-Prot | 33572 | 0.9314 |
| Pfam | 33795 | 0.9376 |
| KEGG | 26304 | 0.7298 |
| GO | 28128 | 0.7804 |
| COG | 34964 | 0.97 |
| Total_anno | 36024 | 0.9994 |
| Total | 36044 | 1 |

Supplementary Table S12 miRNA differential gene number statistics

| diff_group | total DET | up | down |
| --- | --- | --- | --- |
| CK_vs_BCAA | 84 | 45 | 39 |

Supplementary Table S13 GO enrichment analysis (Target mRNAs of Downregulated DEMs)

| GO enrichment analysis(Target mRNAs of Downregulated DEMs) | | | | | | | | |
| --- | --- | --- | --- | --- | --- | --- | --- | --- |
| **Number** | **GO ID** | **Term Type** | **Description** | **Ratio_in_study** | **Ratio_in_pop** | **Pvalue** | | **Padjust** |
| 26 | GO:0048638 | BP | regulation of developmental growth | 26/3489 | 144/40288 | 0.000290120111888 | | 0.203551494057 |
| 19 | GO:0008066 | MF | glutamate receptor activity | 19/3489 | 90/40288 | 0.000219286880457 | | 0.162904353723 |
| 194 | GO:0004672 | MF | protein kinase activity | 194/3489 | 1641/40288 | 0.0000131143663186 | | 0.0207026665297 |
| 115 | GO:0004674 | MF | protein serine/threonine kinase activity | 115/3489 | 861/40288 | 0.00000693631319122 | | 0.0207026665297 |
| 308 | GO:0016772 | MF | transferase activity, transferring phosphorus-containing groups | 308/3489 | 2819/40288 | | 0.0000222782608368 | 0.02736151658 |
| 1133 | GO:0050789 | BP | regulation of biological process | 1133/3489 | 11627/40288 | 0.0000129496613956 | | 0.0207026665297 |

Supplementary Table S14 KEGG enrichment analysis (Target mRNAs of Downregulated DEMs)

| KEGG enrichment analysis(Target mRNAs of Downregulated DEMs) | | | | | | |
| --- | --- | --- | --- | --- | --- | --- |
| **Num** | **Pathway id** | **Description** | **Ratio_in_study** | **Ratio_in_pop** | **Pvalue** | **Padjust** |
| 56 | map04140 | Autophagy - animal | 56/3334 | 440/38306 | 0.00269983727823 | 0.0575402819922 |
| 57 | map04910 | Insulin signaling pathway | 57/3334 | 425/38306 | 0.000739438661463 | 0.0360212262227 |
| 43 | map00190 | Oxidative phosphorylation | 43/3334 | 302/38306 | 0.0009583261312 | 0.0326789210739 |
| 57 | map04371 | Apelin signaling pathway | 57/3334 | 452/38306 | 0.00304856857976 | 0.0611506991587 |
| 100 | map04144 | Endocytosis | 100/3334 | 832/38306 | 0.000635342359886 | 0.0433303489442 |
| 20 | map04710 | Circadian rhythm | 20/3334 | 110/38306 | 0.00122567212145 | 0.0379958357649 |

Supplementary Table S15 GO enrichment analysis (Target mRNAs of Upregulated DEMs)

| GO enrichment analysis(Target mRNAs of Upregulated DEMs) | | | | | | | |
| --- | --- | --- | --- | --- | --- | --- | --- |
| **Number** | **GO ID** | **Term Type** | **Description** | **Ratio_in_study** | **Ratio_in_pop** | **Pvalue** | **Padjust** |
| 564 | GO:0031326 | BP | regulation of cellular biosynthetic process | 564/6070 | 3194/40288 | 0.000036737653959 | 0.0183286101111 |
| 554 | GO:0010556 | BP | regulation of macromolecule biosynthetic process | 554/6070 | 3121/40288 | 0.0000270888515667 | 0.0155726817482 |
| 552 | GO:2000112 | BP | regulation of cellular macromolecule biosynthetic process | 552/6070 | 3108/40288 | 0.0000261214255549 | 0.0155726817482 |
| 1928 | GO:0050789 | BP | regulation of biological process | 1928/6070 | 11627/40288 | 0.0000145793918781 | 0.0140190205646 |
| 776 | GO:0031323 | BP | regulation of cellular metabolic process | 776/6070 | 4396/40288 | 0.000010695181608 | 0.0140190205646 |

Supplementary Table S16 KEGG enrichment analysis (Target mRNAs of Upregulated DEMs)

| KEGG enrichment analysis(Target mRNAs of Upregulated DEMs) | | | | | | |
| --- | --- | --- | --- | --- | --- | --- |
| **Num** | **Pathway id** | **Description** | **Ratio_in_study** | **Ratio_in_pop** | **Pvalue** | **Padjust** |
| 44 | map04115 | p53 signaling pathway | 44/5927 | 203/38306 | 0.0116613331616 | 0.361501328008 |
| 92 | map04141 | Protein processing in endoplasmic reticulum | 92/5927 | 464/38306 | 0.00666207146456 | 0.324538052774 |
| 127 | map05206 | MicroRNAs in cancer | 127/5927 | 628/38306 | 0.000792956232018 | 0.270398075118 |
